# Quorum Quenching with a Diffusible Signal Factor Analog in *Stenotrophomonas maltophilia*

**DOI:** 10.3390/pathogens12121448

**Published:** 2023-12-14

**Authors:** Dafne Guillén-Navarro, Rosa González-Vázquez, Gloria León-Ávila, Silvia Giono-Cerezo

**Affiliations:** 1 Escuela Nacional de Ciencias Biológicas, Instituto Politécnico Nacional, Departamento de Microbiología, Prolongación de Carpio y Plan de Ayala S/N, Col. Casco de Santo Tomás, Alcaldía Miguel Hidalgo, Mexico City 11340, Mexico; 2 Posgrado en Ciencias Quimicobiológicas, Escuela Nacional de Ciencias Biológicas, Instituto Politécnico Nacional, Prolongación de Carpio y Plan de Ayala S/N, Col. Casco de Santo Tomás, Alcaldía Miguel Hidalgo, Mexico City 11340, Mexico; 3 Instituto Mexicano del Seguro Social, Unidad Médica de Alta Especialidad, Hospital de Especialidades “Dr. Antonio Fraga Mouret”, Centro Médico Nacional La Raza. Seris y Zaachila S/N, Col. La Raza, Alcaldía Azcapotzalco, Mexico City 04960, Mexico; 4 Escuela Nacional de Ciencias Biológicas, Instituto Politécnico Nacional, Departamento de Zoología, Prolongación de Carpio y Plan de Ayala S/N, Col. Casco de Santo Tomás, Alcaldía Miguel Hidalgo, Mexico City 11340, Mexico

**Keywords:** Quorum Quenching, Quorum Sensing, diffusible signal factor, MLST, retinoids

## Abstract

*Stenotrophomonas maltophilia* is a multidrug-resistant Gram-negative bacillus associated with nosocomial infections in intensive care units, and nowadays, its acquired resistance to trimethoprim–sulfamethoxazole (SXT) by *sul* genes within class 1 integrons is a worldwide health problem. Biofilm and motility are two of the major virulence factors in this bacterium and are auto-induced by the diffusible signal factor (DSF). In recent studies, retinoids have been used to inhibit (Quorum Quenching) these virulence factors and for their antimicrobial effect. The aim was to reduce biofilm formation and motility with retinoic acid (RA) in *S. maltophilia* SXT-resistant strains. Eleven SXT-resistant strains and two SXT-susceptible strains were tested for biofilm formation/reduction and planktonic/sessile cell viability with RA and SXT-MIC_50_/RA; motility (twitching, swimming, swarming) was measured with/without RA; and MLST typing was determined. The biofilm formation of the strains was classified as follows: 15.38% (2/13) as low, 61.54% (8/13) as moderate, and 23.08% (3/13) as high. It was significantly reduced with RA and SXT-MIC_50_/RA (*p* < 0.05); cell viability was not significantly reduced with RA (*p* > 0.05), but it was with SXT-MIC_50_/RA (*p* < 0.05); and swimming (*p* < 0.05) and swarming (*p* < 0.05) decreased significantly. MLST typing showed the first and novel strains of Mexican *S. maltophilia* registered in PubMLST (ST479-485, ST497, ST23, ST122, ST175, ST212, and ST300). In conclusion, RA reduced biofilm formation and motility without affecting cell viability; furthermore, antimicrobial synergism with SXT-MIC_50_/RA in different and novel STs of *S. maltophilia* was observed.

## 1. Introduction

*Stenotrophomonas maltophilia* is a multidrug-resistant bacterium treated with SXT, and according to the Clinical and Laboratory Standards Institute (CLSI), there are reports of resistance to SXT attributed to the *sul*1 and *sul*2 genes [1], which are codified as dihydropteroate synthase and interrupted folic acid synthesis [2]. They are at the 3′ end of class 1 integrons within the chromosome or plasmids and confer horizontal gene transference of SXT [3].

*S. maltophilia* produces biofilms, which are microorganism communities within the matrix of an extracellular polysaccharide, and this is considered a risk factor during patient recovery [4]. Biofilm-mediated resistance to antibiotics has been specified as being a result of (1) a matrix that acts as a diffusion barrier, (2) the limitation of nutrients in the microenvironment, which reduces antimicrobial activity, (3) the metabolic inactivity of persistent cells, (4) chemical interactions between antimicrobials and biofilm components, (5) horizontal gene transfer allowing for the acquisition of resistance genes, and (6) nutrients released from dead cells after the use of antibiotics, which favor cell growth in inner layers [4,5,6].

Quorum Sensing (QS) is cell-to-cell signalization produced by autoinducers (AI) synthesized at high cell concentrations, in which membrane or cytoplasmic receptors detect them and generate a growth, motility, and biofilm formation response [7]. This stimulates cell synchronization, horizontal gene transference, and interactions with different species of microorganisms [8]. QS in *S. maltophilia* is established by DSF (11-metyl cis-2-dodecenoic acid) fatty acid signaling, which regulates swimming, swarming, and biofilm formation. The cis-double bond between C2 and C3 is an essential requirement for DSF activity [9].

Quorum Quenching (QQ) participates in QS interruption; the main point is not to cause cell death due to the low selective pressure that it generates [6]. The most widely used strategy to generate QQ in *S. maltophilia* is with AI analogs to DSF; these can be synthetic, synthesized by other microorganisms or by complex organisms, such as plants [9,10]. Reviews on QQ systems in Gram-negatives describe those generated by enzymes (lactonases, acylases, and oxidoreductases) and inhibitors/analogs [11]. So far, there are no published QQ studies on the DSF-QS system in *S. maltophilia;* certain publications only mention it [12].

Sulfonamide-based bioisosteres are used as DSF analogs because of their high physicochemical similarity. They interrupt QS signaling [9], for example, in *Pseudomonas aeruginosa* and *Burkholderia cepacia*, inhibit biofilm formation and swarming [13], and have bactericidal activity against *Enterococcus faecalis* [14]. Bioisosteres as retinoids have properties attributed to cell differentiation in embryonic development and, recently, as treatment against cancer since they induce apoptosis in cancerous cells without affecting healthy cells [15,16].

RA is an active metabolite of vitamin A obtained as a preformed vitamin A (retinol and retinyl esters). Liver dehydrogenases convert retinol into RA [17], which has three natural isomers—9-cis, 13-cis, and all-trans-retinoic acid (ATRA)—that have shown different cellular properties and different affinities to receptors. They also have immunomodulatory capacities to cause effects on B-cell responses, are cell- and tissue-dependent, and regulate the interface between innate and adaptative responses. Recent studies have shown that ATRA also has an antitumoral role in solid cancers, such as gastric cancer, and it is used as a treatment against promyeloid leukemia given its remission rate from 15% to 85% [18,19,20]. During infections, RA induces the production of pro-inflammatory cytokines by dendritic cells, promotes the differentiation of effector T cells, and protects the mucosa because it is crucial for maintaining homeostasis at the intestinal barrier and equilibrating immunity and tolerance [21].

ATRA also has fungicidal and fungistatic activity; affects *Candida* spp. biofilm formation in terms of biomass, morphology, and metabolic activity; is dose-dependent; and has synergy with amphotericin B [17,21]. RA could be considered an analog of DSF due to the unsaturated cis-double bond between C2 and C3, which disrupts the recognition of DSF by the ten transmembrane regions possessed by RpfC, and for the different substituents, which change the conformation compared to those of DSF [9]. Therefore, differences in chain size and substituents affect the synthesis of virulence factors [15].

Another molecule that has been evaluated on biofilm formation is ascorbic acid, which is a non-chemotherapeutic alternative that suppresses biofilm formation without causing resistance [22], since it inhibits Quorum detection. At 30 mM, ascorbic acid exposes the bacteria to the medium, making them more susceptible to death due to oxidative stress [23].

In this study, we analyzed the use of RA to reduce biofilm formation and motility in *S. maltophilia* SXT-resistant strains by means of mixed antibiotics therapy in different STs.

## 2. Materials and Methods

### 2.1. Strains of Stenotrophomonas maltophilia

Thirteen strains of *S. maltophilia* were selected for this study. The strains were registered at CnRGM-INIFAP and collected from different hospitals in Mexico City. They were phenotypically re-identified using an automated Vitek^®^ 2 system (BioMérieux, Marci l’Étoile, France) and kept at −70 °C in brain–heart infusion broth (BHI, BD-Difco, Franklin Lakes, NJ, USA) supplemented with 10% glycerol (Sigma-Aldrich, St. Louis, MO, USA).

### 2.2. Trimethoprim-Sulfamethoxazole Susceptibility Test

The minimal inhibitory concentration (MIC) of SXT (Sigma-Aldrich, MO, USA) was assessed according to CLSI, 2020 [24]. The microdilution method in Mueller–Hinton cation-adjusted broth (MHCAB, BD-Difco, MD, USA) was used to determine the MIC. *Escherichia coli* ATCC 25922 and *S. maltophilia* 17666 were used as controls. Cutoff point: susceptible (S), MIC ≤ 2/38 µg/mL; resistant (R), MIC ≥ 4/76 µg/mL.

### 2.3. Detection of Resistance Genes

The integrons of class 1 (*int*I-1), class 2 (*int*I-2), and class 3 (*int*I-3) and sulfonamide (*sul*1 and *sul*2) genes were amplified via polymerase chain reaction (PCR); the primers used are mentioned in Table 1.

PCR cycling parameters were as follows: 94 °C for 5 min, followed by 30 cycles of 94 °C for 15 s, 64 °C for 30 s (*int*I-1), 62 °C for 30 s (*sul*1), or 57 °C for 30 s (*sul*2), and 72 °C for 1 min, ending with 72 °C for 5 min. For *int*I-2 and *int*I-3, the PCR cycle parameters were as follows: 95 °C for 5 min, followed by 30 cycles of 95 °C for 1 min, 62 °C for 1 min, and 72 °C for 50 sec, ending with 72 °C for 5 min. The PCR assays for the detection of the acquired resistance genes were performed in triplicate.

DNA extraction was performed with the guanidinium thiocyanate method [29]. *Aeromonas taiwanensis* PIM6 was used as a positive control for *int*I-2.

### 2.4. Multilocus Sequence Typing

MLST typing was assayed according to Kaiser, 2009 [30]. Seven housekeeping genes (*atp*D, *gap*A, *gua*A, *mut*M, *nuo*D, *ppsA*, and *rec*A) were amplified, sequenced, and analyzed with the PubMLST database to identify the allelic profiles, and a sequence type (ST) was assigned for each strain. *S. maltophilia* ATCC 17666 was used as a positive control. To identify and visualize the relationship within isolates, we used PHYLOViZ v2.0 [31]. Phylogenetic analyses of each strain’s housekeeping sequences were concatenated and aligned in MEGA11 [32] using a UPGMA dendrogram with the TN93 + G model.

### 2.5. Biofilm Formation and Reduction

Biofilm formation was assessed using the crystal violet (CV) staining method, as previously described by Stepanovic et al., 2007 [33], with a few modifications. The *S. maltophilia* colonies were collected and diluted in BHI broth and grown overnight. The suspension was adjusted to 0.5 MacFarland (1 × 10^8^ cells/mL) and incubated at 37 °C for 24 h under static conditions. The samples were washed three times with PBS, and then the cells were fixed at 60 °C for 1 h. After 200 µL of CV 0.1% was added to the wells, the samples were incubated for 20 min at room temperature and washed three times with PBS before adding 200 µL of ethanol (100%) for 20 min. The assays were performed in triplicate in 96-well microplates, *Staphylococcus aureus* ATCC 25923 (positive), a *Rhodococcus equi* strain (negative), *S. maltophilia* ATCC 17666 (cell), and no inoculated BHI broth (medium) were used as controls. Biofilm formation was measured on a spectrophotometer (MultiskanTM FC, ThermoFisher Scientific Inc, Waltham, MA, USA) at 620 nm. The mean (A) and standard deviation (SD) were calculated for each assay. The cutoff absorbance (Ac) was defined as the value of three times the SD above the A of the negative control (Ac = A of the negative control + 3 SD of the negative control) [34]. The strains were classified into four categories based on the Ac and the A: no producers (A ≤ Ac), low producers (Ac < A ≤ 2 Ac), moderate producers (2 Ac < A ≤ 4 Ac), and high producers (4 Ac < A) [34]. For the biofilm reduction assay, RA 0.2 M (Sigma Aldrich, San Luis, MO, USA) SXT-MIC_50_, and SXT-MIC_50_/RA were added. The biofilm reduction quantification methodology was the same, only modified for an initial inoculum of 100 µL at 0.5 MacFarland, and 100 µL of the mix was used for biofilm reduction [35].

### 2.6. Motility and Motility Reduction Test

Twitching, swimming, and swarming tests were performed in triplicate, according to Rashid and Kornberg, 2000 [35]. For each trial, the average displacement distance, A, and SD were calculated. The reduction in motility was to determine whether RA also decreased it. The methodology was similar; the only difference was the addition of 20 µL of RA 0.2 M at the inoculation point, and then the displacement was measured.

### 2.7. Viability Test

Viability tests were carried out in triplicate using microdilutions in 96-well microplates according to Tan et al., 2019 [14]. The following variables were used: untreated, RA, SXT-MIC_50_, and SXT-MIC_50_/RA. CFU/mL was counted and averaged, calculating the A and SD.

### 2.8. Statistical Analysis

Student’s *t*-test was used for comparison between groups, one-way ANOVA was used for comparisons of multiple groups, and StatView v4.5 for Windows (www.statview.com; accessed on 11 August 2021) was used to determine the statistical significance of the data. A *p-*value of <0.05 was considered to indicate statistical significance.

## 3. Results

### 3.1. Trimethoprim–Sulfamethoxazole Susceptibility Test

The MIC via SXT microdilution was measured according to the CLSI, 2020 [24]. The strains were identified as 84.62% (11/13) resistant to SXT and 15.38% (2/13) susceptible to SXT (Table 2).

### 3.2. Detection of Resistance Genes

Of the thirteen *S. maltophilia* strains, only those strains classified as SXT-resistant via the MIC method were amplified for *int*I-1, *sul*1, and *sul*2 genes. No strain was amplified for the *int*I-2 and *int*I-3 genes.

### 3.3. Multilocus Sequence Typing

**Figure 1:**
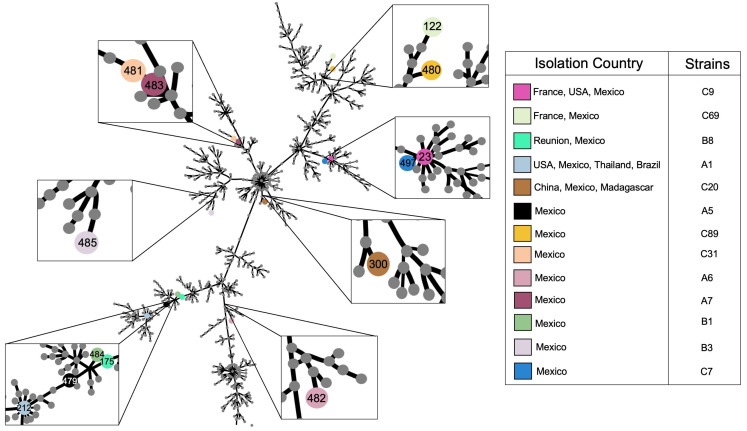
eBURST ST population from *S. maltophilia*. There are 2217 STs registered (until 4 December 2023) in the PubMLST database, and the STs obtained are distributed throughout the tree. The registered STs are represented with differently colored dots. There are two ST founders of clonal complexes (ST23 and ST212). eBURST was performed based on single locus variants (SLV) on the PHYLOViZ web server [31].

Analysis of MLST showed that the strains had different and novel STs. We sent the sequences to the PubMLST database for revision, and they assigned new STs to eight strains. All STs were registered in the database and were the first ones registered from Mexico (Figure 1).

**Figure 2:**
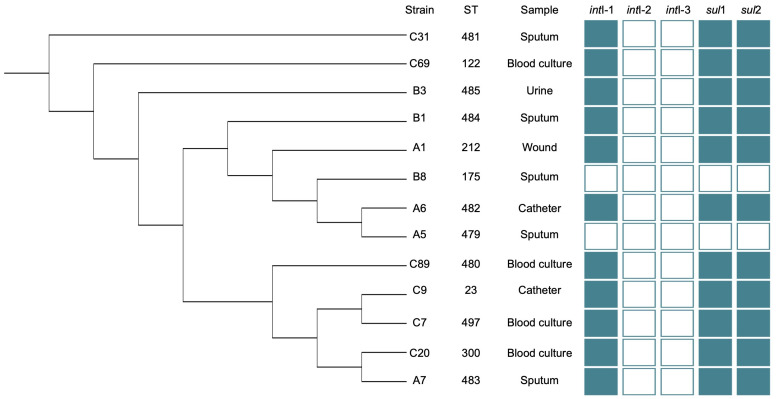
Concatenated UPGMA dendrogram of *S. maltophilia* housekeeping genes. The dendrogram shows the distribution of the strain according to its allelic profile. Only four STs are closely related (A5 and A6; C7 and C9). ST and sample information is given, as well as the presence/absence of the acquired resistance genes. Visualization was carried out using iTOL v6 [36].

An UPGMA dendrogram was built with the concatenated sequences of the seven housekeeping genes used (Figure 2), and they were analyzed using the TN93 + G model.

The UPGMA dendrogram shows that the strains are not distributed in clades according to the isolation place, but they are distributed throughout the tree and the STs already registered.

### 3.4. Biofilm Formation and Reduction Assay

According to the cutoff absorbance of the biofilm formation assays and the cutoff absorbance (Ac = 0.100) of the negative control, the strains were classified into null (≤0.100), low (0.101–0.199), moderate (0.200–0.399), and high producers (≥0.400). According to these parameters, the classification was as follows: 15.38% (2/13) as low, 61.54% (8/13) as moderate, and 23.08% (3/13) as high producers (Figure 1).

**Figure 3:**
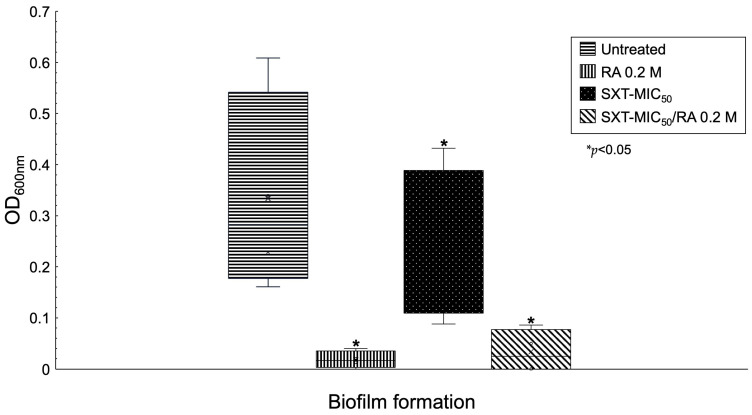
Comparison of biofilm formation by *S. maltophilia* with different treatments. The reduction in biofilms depended on the compound added. When RA and SXT-MIC_50_/RA were added, biofilm formation decreased significantly (*p* < 0.05).

Biofilm reduction assays were performed with each variable mentioned above in triplicate. Null biofilm formation was obtained by adding RA 0.2 M and SXT-MIC_50_/RA (*p* < 0.05). Treatment with SXT-MIC_50_ significantly reduced biofilm formation to moderate (*p* < 0.05). (Figure 3).

### 3.5. Motility and Interruption Assay

**Figure 4:**
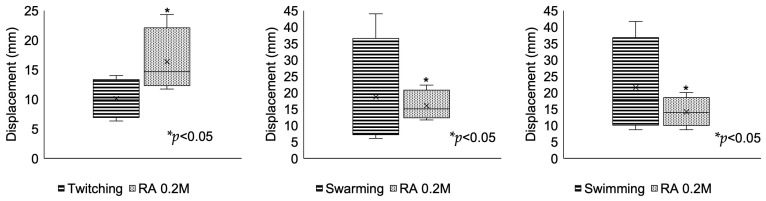
Motility assays of twitching, swimming, and swarming, and their reduction. The strains had more significant displacement through swarming and swimming. When we added RA, motilities through swarming (*p* < 0.05) and swimming (*p* < 0.05) decreased significantly. The motility through twitching increased significantly with the addition of RA (*p* < 0.05).

The strains had predominant swimming and swarming motilities, mainly associated with DSF-QS [9]. With the addition of RA, swimming (*p* < 0.05) and swarming (*p* < 0.05) decreased significantly. In the case of twitching, it was observed that this was the lowest, and it behaved in a different way from the other two motilities in the presence of RA, as the motility increased significantly (*p* < 0.05) (Figure 4).

### 3.6. Viability Assay

QS inhibitors do not kill microorganisms; they remain viable [37]. Cell viability assays were performed in triplicate, counting CFU/mL from untreated cells, with RA, SXT-MIC_50_, and SXT-MIC_50_/RA.

**Figure 5:**
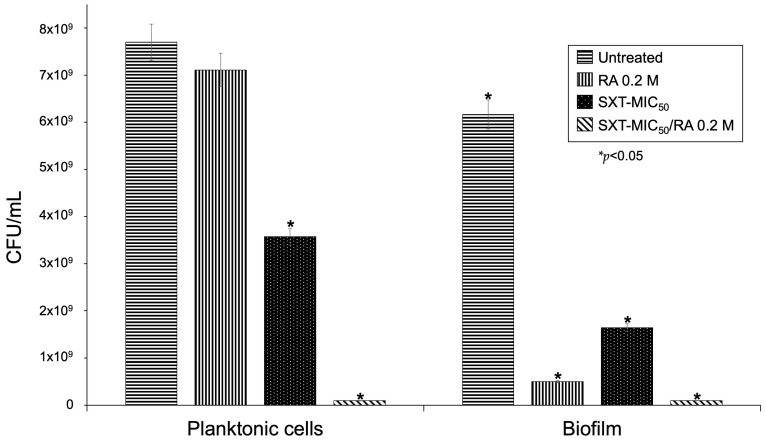
Viability assays of planktonic and sessile cells of *S. maltophilia* treated with SXT and RA. In planktonic cells, the addition of RA 0.2 M did not lead to a significant difference in CFU/mL (*p* > 0.05), but with the addition of SXT-MIC_50_ and SXT-MIC_50_/RA, the viability was reduced significantly (*p* < 0.05). In planktonic cells and biofilm, the addition of RA to the biofilm significantly reduced its formation (*p* < 0.05).

RA treatment did not affect the viability of planktonic and sessile cells (*p* > 0.05), although, in sessile cells, the CFU/mL was low because there was no biofilm formation, and they were dispersed in the medium. Treatment with SXT-MIC_50_ decreased the viability in planktonic and sessile cells (*p* < 0.05) since it was the concentration to which 50% of the strains were susceptible. In the presence of SXT-MIC_50_/RA, the viability decreased (*p* < 0.05). We consider that RA and SXT could be acting synergistically (Figure 5).

## 4. Discussion

*S. maltophilia* is an emerging and opportunistic pathogen related to healthcare-associated infections (HAIs) and is one of the most frequently isolated microorganisms [37].

*S. maltophilia* is considered an MDR pathogen treated with SXT; however, there are several reports of resistance to SXT by class 1 integrons, in which *sul*1 and *sul*2 sulfonamide resistance genes are found [3]. In future studies, it will be necessary to sequence the integrons to know their conformation and location, since they could be in plasmids or in bacterial genophore. In Mexican strains of *S. maltophilia,* there is only one report of class 2 integrons [38], while class 3 integrons have not been detected yet; several authors recommend designing primers specifically for *S. maltophilia* integrons [39,40].

The MLST technique analyzes global long-term epidemiology, for which it is necessary to know the population structure of samples from different geographical areas and year collections [41]. In this study, strains C7 and C9 were isolated from the same sample and presented differences in the *atp*D allele; therefore, C7 was identified as a different and new ST. This suggests that due to the microenvironmental characteristics of the isolated samples and their respective selective pressures, the strains were adapted and generated differences in the nucleotide arrangement or that the patient developed a co-infection with different STs, as reported in 2017 by Esposito et al. in patients with cystic fibrosis [42].

Strain A5 was reported as SXT susceptible compared to strain A6, and both were isolated from the same zone and showed differences in the *mut*M and *nuo*D alleles, which generated different and novel STs. The *mut*M gene encodes the synthesis of DNA formamide-pyrimidine glycosylase, which is the first line of defense against highly mutagenic damage, such as that caused by oxidative stress from radical oxygen species (ROS), especially by antimicrobials such as SXT [43].

Biofilm formation is one of the virulence factors of *S. maltophilia* that contributes to resistance; this increases the difficulty of patient recovery by up to 80% [6]. DSF is the most studied signaling system for biofilm formation and motility in *S. maltophilia* [9]. It was described for the first time in *Xanthomonas campestris* pv. *Campestris*, but it is not exclusive to Xantomonadales [44] or other species, such as *Burkholderia cepacia* (BDSF) and *P. aeruginosa* (PDSF), whose family of lipophilic molecules has an unsaturated double bond between C2 and C3, close to carboxylic acid, which was recognized among species possessing this same type of QS signaling [7,9].

*S. maltophilia* mechanisms of QQ have not been specified; instead, the use of DSF-QS analogs as bioisosteres of the p-aminobenzoic acid substrate, on which sulfonamides act, was observed to reduce biofilm formation and MIC of colistin. Combined with this, it was observed that there is a bactericidal activity [10]. Retinoid-derived bioisosteres have also been used [11,45]. RA is a bioisoster that has been tested in vitro and used as a treatment against Gram-positive bacteria, as it also presents synergy when combined with gentamicin. In this model, antibiofilm activity and motility were obtained via swarming, determining that the antimicrobial synergism activity was due to RA breaking the cell membrane, which allowed for gentamicin entry into the cell [15].

RA was the compound selected for the reduction in biofilm formation; it was shown that when RA 0.2 M was added to the culture medium, the biofilm formation decreased significantly (*p* < 0.05), which would reclassify all the strains as non-producers (Figure 3).

The formation of biofilms in strains is not related to SXT-MIC. In SXT-resistant strains, there have been no studies on whether there is a way to inhibit biofilm formation; there is only one reference for the absence of biofilm formation at a high concentration of SXT (500 µg/mL), although this can be counterproductive, as it increases the probability of acquiring resistance to SXT [34].

According to the results, by adding SXT-MIC_50_ to the culture, biofilm formation was reduced, even in SXT-resistant strains. RA could also be considered a candidate DSF analog since it has the characteristics described by Huedo et al., 2019 [10]. It can be recognized by RpfC, conserving the unsaturated double bond in C2 and C3. The chain length and substituents influence whether the auto-inducer is recognized [45].

Biofilm formation did not correlate with the CFU/mL results obtained, which is attributed to the bacterial lysis generated due to higher density and dispersion, attributable to the biofilm components dispersed in the medium, suggesting that SXT-MIC_50_ acts synergistically with RA. Fluorescence or atomic force electron microscopy would be necessary to correlate biofilm production with viability [46,47], or the use of matrix-assisted laser desorption ionization/time-of-flight, looking for the different biomarker peaks [48].

Nowadays, multiple efforts have been made in research to propose new treatments. QQ is one of them, mainly because of the promise of not generating resistance to antibiotics due to its low selective pressure. Although *S. maltophilia* is an important pathogen, the knowledge about RA is limited. Some bioisosteres have been tested, with promising results; however, more research is needed to understand the mechanisms of action of them. 

## 5. Conclusions

Multiple efforts have been made to find new treatments for HAIs. A better understanding of the mechanisms involved in biofilm formation in *S. maltophilia* will contribute to the discovery of novel alternatives with greater efficacy against MDR pathogens. In this study, we observed that retinoic acid (a vitamin A metabolite, also known as retinol) and SXT-MIC_50_/RA did not affect cell viability; they reduced biofilm formation and the motility of *S. maltophilia*; and they also had synergistic activity. SXT resistance was related to the presence of genes *int*I-1, *sul*1, and *sul*2.

Retinoic acid worked against different STs of *S. maltophilia*. Since distinct STs were identified and registered for the first time in Mexico, the importance of molecular epidemiology is emphasized.

## Figures and Tables

**Table 1:** Primers used to detect resistance genes.

Gene	Primer ^1^	Sequence 5′–3′	Molecular Size (pb)	Reference
*int*I-1	intI1-F intI1-R	TCATGGCTTGTTATGACTGT GTAGGGCTTATTATGCACGC	580	[25]
*int*I-2	intI2-F int2-R	CACGGATATGCCACAAAAAGGT GTAGCAAACGAGTGACGAAATG	240	[26]
*int*I-3	intI3-F int3-R	AGTGGGTGGCGAATGAGTG TGTTCTTGTATCGGCAGGTG	758	[27]
*sul*1	sul1-F sul1-R	ATGGTGACGGTGTTCGGCATTCTGA CTAGGCATGATCTAACCCTCGGTCT	900	[28]
*sul*2	sul2-F	GCGCTCAAGGCAGATGGCATT	293	[2]
	sul2-R	GCGTTTGATACCGGCACCCGT		

^1^ All primers were at a 1:10 dilution for the PCR mix.

**Table 2:** Minimum inhibitory concentrations of trimethoprim/sulfamethoxazole in *Stenotrophomonas maltophilia* strains ^1^.

Strains	Trimethoprim/Sulfamethoxazole MIC (µg/mL)	Susceptibility
A1	16/304	R
A5	2/38	S
A6	16/304	R
A7	16/304	R
B1	16/304	R
B3	8/152	R
B8	0.25/4.75	S
C7	32/608	R
C9	32/608	R
C20	16/304	R
C31	16/304	R
C69	16/304	R
C89	16/304	R
*S. maltophilia* ATCC 17666	16/304	R

^1^ Cutoff point: susceptible (S), MIC ≤ 2/38 µg/mL; resistant (R), MIC ≥ 4/76 µg/mL.

## Data Availability

All relevant data have been included in this manuscript.
